# The Effects of Reactant Concentration and Air Flow Rate in the Consumption of Dissolved O_2_ during the Photochemistry of Aqueous Pyruvic Acid

**DOI:** 10.3390/molecules24061124

**Published:** 2019-03-21

**Authors:** Alexis J. Eugene, Marcelo I. Guzman

**Affiliations:** Department of Chemistry, University of Kentucky, Lexington, KY 40506, USA; alexis.eugene@uky.edu

**Keywords:** pyruvic acid, dissolved O_2_, photolysis, SOA

## Abstract

The sunlight photochemistry of the organic chromophore pyruvic acid (PA) in water generates ketyl and acetyl radicals that contribute to the production and processing of atmospheric aerosols. The photochemical mechanism is highly sensitive to dissolved oxygen content, [O_2_(*aq*)], among other environmental conditions. Thus, herein we investigate the photolysis (λ ≥ 305 nm) of 10–200 mM PA at pH 1.0 in water covering the relevant range 0 ≤ [O_2_(*aq*)] ≤ 1.3 mM. The rapid consumption of dissolved oxygen by the intermediate photolytic radicals is monitored in real time with a dissolved oxygen electrode. In addition, the rate of O_2_(*aq*) consumption is studied at air flow rates from 30.0 to 900.0 mL min^−1^. For the range of [PA]_0_ covered under air saturated conditions and 30 mL min^−1^ flow of air in this setup, the estimated half-lives of O_2_(*aq*) consumed by the photolytic radicals fall within the interval from 22 to 3 min. Therefore, the corresponding depths of penetration of O_2_(*g*) into water (*x* = 4.3 and 1.6 µm) are determined, suggesting that accumulation and small coarse mode aqueous particles should not be O_2_-depleted in the presence of sunlight photons impinging this kind of chromophore. These photochemical results are of major tropospheric relevance for understanding the formation and growth of secondary organic aerosol.

## 1. Introduction

Pyruvic acid (PA, p*K_a_* = 2.39) [1] is an abundant component of tropospheric aerosols [2,3,4], and one of the few chromophores capable of undergoing photochemical reactions in water [5,6,7,8,9,10]. It is produced during the atmospheric photooxidative processing of biogenic and anthropogenic emissions [11,12]. If formed in the gas phase, PA preferentially partitions into the aqueous phase with a large Henry’s law constant (*K*_H_ = 3.1 mol kg^−1^ Pa^−1^) [13], and a reactive uptake coefficient by water of $\gamma_{PA}$ = 0.06 [14]. During this uptake, the interfacial accommodation process for gaseous PA onto the surface of water implies the contribution of a plane parallel orientation of the –COOH, C=O, and –CH_3_ groups to the interface [14,15]. In addition, the C=O group of PA undergoes cooperative reversible hydration (with equilibrium constant, *K*_Hyd_ = 2.10 at 25 °C [16]) into 2,2-dihydroxypropanoic acid, a UV transparent gem-diol [17,18,19,20]. Early studies of the photochemistry of PA reported that for a fraction of pyruvate larger than 0.99 (at pH 6.1), the reactant decays at least an order of magnitude slower than for the undissociated acid [21]. A more recent study reported qualitative observations on the effect of pH during photolysis [22] consistent with earlier work.

**Scheme 1:**
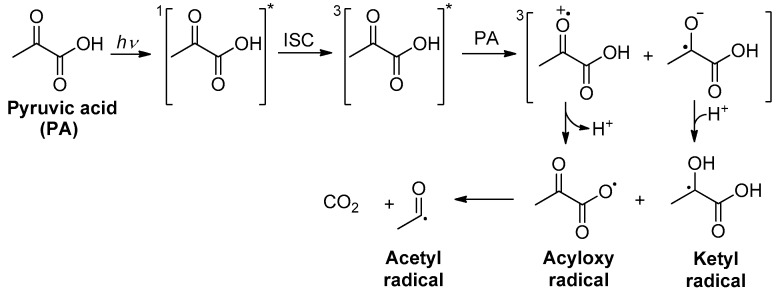
Initial proton-coupled electron transfer step for the photochemistry of aqueous pyruvic acid (PA). Adapted from ref. [5].

The processing of PA in atmospheric waters by sunlight’s photons [7,8,9,23,24,25,26] and by hydroxyl radicals (HO^•^), generated from the photolysis of H_2_O_2_ [11], have also been reported. Upon excitation of PA, the produced excited state singlet ^1^PA^*^ quickly undergoes intersystem crossing (ISC) to produce a reactive triplet ^3^PA^*^. This triplet reacts with a ground state molecule of PA as described in Scheme 1. The proton-coupled electron transfer (PCET) mechanism in Scheme 1 is based on photochemistry studies of aqueous pyruvic acid that trapped radical pairs during the λ = 320 (±10) nm photolysis of frozen solutions at −196 °C [5,27]. These radical pairs were characterized by electron paramagnetic resonance (EPR) spectroscopy indicating that ketyl (K^•^) and acetyl (Y^•^, which is quickly hydrated) radicals formed with unpaired spins separated by ≥ 0.5 nm [5]. In Scheme 1, the acyloxy radical is a metastable species that decarboxylates exothermically forming the acetyl radical and making the overall energetics of the reaction favorable. Moreover, the EPR signals of the radical pairs were stable toward temperature changes below −93 °C. However, upon warming above the sublimation temperature of CO_2_ (−133 °C), the frozen sample began to release appreciable amounts of CO_2_, indicating a unidirectional process proceeded after no more forced close contact of radical pairs was kept [5]. Overall, the photogeneration of these radicals is summarized as represented in Scheme 1. Even though other viewpoints could exist for the initial steps, they key common point among all proposals is that ketyl and acetyl radicals are photoproduced from PA [7,8].

A subject of major interest has been the use of the photogenerated acetyl and ketyl radicals from irradiated PA solutions to initiate the processing of other water soluble organic atmospheric compounds. For example, the oligomerization of methyl vinyl ketone (MVK) [28], an important water-soluble intermediate arising from the atmospheric oxidation of isoprene [29], proceeds in the presence of irradiated aqueous PA. The effect of dissolved oxygen content, [O_2_(*aq*)], in the previous work has been described to be detrimental to the oligomerization of MVK [28]. The light triggered experiments with solutions containing PA and MVK were run in a batch mode with an initial fixed [O_2_(*aq*)] = 0.26 mM [28]. However, no effort to replenish the consumed amount of O_2_(*aq*) was attempted in the work with MVK and PA [28]. Such extreme conditions were conducive to quickly consuming all dissolved O_2_ by the generated radicals of PA photolysis. Therefore, this work explores for the first time the experimental conditions for the generation of photolytic radicals of atmospheric relevance under realistic environmental conditions. The experiments below aim to study in detail the dependence of the consumption rate of [O_2_(*aq*)] by the photolytic radicals generated using (1) variable initial [PA]_0_, (2) a range of flow rates for sparging air continuously, and (3) generated mixtures of O_2_(*g*) in N_2_(*g*) to reach the saturation conditions for [O_2_(*aq*)] expected under thermodynamic equilibrium.

## 2. Results and Discussion

**Figure 1:**
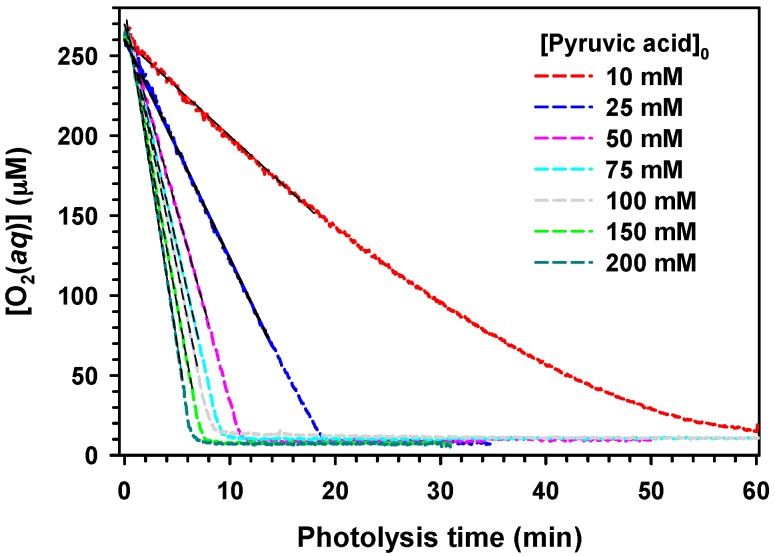
Dissolved oxygen consumption during the photolysis at λ ≥ 305 nm (incident photon rate: *I_0_* = 1.13 × 10^−5^ Einstein L^−1^ s^−1^) of 10.0 ≤ [PA]_0_ ≤ 200.0 mM (as color coded in the figure) at pH 1.0 and 25 °C under continuous air sparging of 30.0 mL min^−1^. The absorbed photon rates are *I_a,10 mM_* = 7.80 × 10^−6^ Einstein L^−1^ s^−1^ for [PA]_0_ = 10 mM, *I_a,25 mM_* = 1.07 × 10^−5^ Einstein L^−1^ s^−1^ for [PA]_0_ = 25 mM; and *I_a,50–200 mM_* = 1.13 × 10^−5^ Einstein L^−1^ s^−1^ for 50 ≤ [PA]_0_ ≤ 200 mM. Representative nonlinear fittings for the zero-order reaction are provided with thin black dash traces.

The first part of this work explores the effect of varying the initial concentration of PA ([PA]_0_) on the consumption of [O_2_(*aq*)]. Figure 1 shows the decay of [O_2_(*aq*)] in experiments with 10.0 ≤ [PA]_0_ ≤ 200.0 mM at pH 1.0 and 25 °C irradiated at λ ≥ 305 nm with an incident photon rate *I_0_* = 1.13 × 10^−5^ Einstein L^−1^ s^−1^. The results in Figure 1 clearly reflect a variation for the limited mass transfer of O_2_(*g*) to the solution. Remarkably, the experiments in Figure 1 exhibit the effect of creating gas bubbles of air that cannot completely recover complete O_2_(*aq*) saturation levels throughout the irradiation period of 1 h. The decay of dissolved oxygen corresponds to complete depletion of O_2_(*aq*) after 6 min and 1 h irradiation for the limit cases studied of 200.0 and 10.0 mM PA, respectively. It is evident in Figure 1 that for higher [PA]_0_, the consumption of O_2_(*aq*) is accelerated until it levels off. Instead, for the lowest [PA]_0_, there is a delay for the first-order decay to reach complete O_2_(*aq*) depletion.

**Figure 2:**
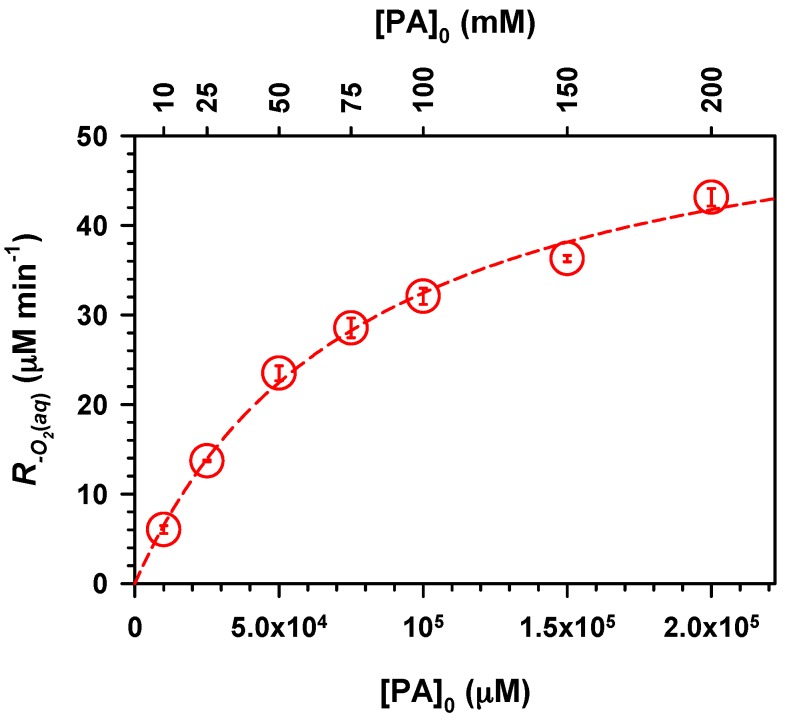
Rate of dissolved oxygen decay ($R_{-O_{2}\left(aq\right)}$) at variable initial concentration of PA ([PA]_0_) during irradiation of solutions at pH 1.0 and 25 °C under continuous air sparging at a flow rate of 30.0 mL min^−1^. The absorbed photon rates are provided in Figure 1. The presented reaction rates are multiplied by −1.

The O_2_(*aq*) decay rates ($R_{-O_{2}\left(aq\right)}$) are obtained from the slopes of the measurements in Figure 1, corresponding to experiments starting with the same concentration of dissolved O_2_. For the range of 25 ≤ [PA]_0_ ≤ 200 mM, the slopes do not change from the beginning to the end of the reaction, when O_2_(*aq*) can be considered to be depleted. The exemption to this observation for [PA]_0_ = 10 mM, which loses its linearity after 25 min of irradiation, is solved by using the initial data to obtain a reaction rate with the same dimensions of a zero order reaction. The negative slopes extracted from Figure 1 are multiplied by −1 to plot the magnitude of the reaction rates, $R_{-O_{2}\left(aq\right)}$, in Figure 2. The Langmuir isotherm type of dependence for $R_{-O_{2}\left(aq\right)}$ on [PA]_0_ in Figure 2 is fitted with a coefficient of correlation *r^2^* = 0.993 by the hyperbola:

(1) $$R_{-O_{2}\left(aq\right)}=\frac{R_{-O_{2}\left(aq\right),max}{\left[\mathrm{PA}\right]}_{0}}{K_{L}+{\left[\mathrm{PA}\right]}_{0}}$$

The nonlinear fitting parameter for the maximum O_2_(*aq*) decay rate, $R_{-O_{2}\left(aq\right),max}$ = 58.6 μM min^−1^, is predicted for a high enough [PA]_0_ to saturate with radicals K^•^ and Y^•^ the surface of the air bubbles. The constant *K_L_* = 8.03 × 10^4^ µM corresponds to the [PA]_0_ needed to cover half of the surface of bubbles available with K^•^ and Y^•^ radicals for the air flow and absorbed photon rates in the experiments.

The integration of Equation (1) yields the solution that perfectly describes the concentration of dissolved O_2_ vs. time (*t*):

(2) $${\left[O_{2}\left(aq\right)\right]}_{t}={\left[O_{2}\left(aq\right)\right]}_{0}-\frac{R_{-O_{2}\left(aq\right),max}{\left[\mathrm{PA}\right]}_{0}}{K_{L}+{\left[\mathrm{PA}\right]}_{0}}t$$

As a demonstration, the zero-order process for all [PA]_0_ in Figure 1 is fitted with the dashed black traces by using the nonlinear regression Equation (2) in Figure 1.

Indeed, the behavior observed in Figure 2 reflects how large [K^•^] and [Y^•^] are produced by photolysis to quickly react with O_2_ at the surface of the air bubbles created. The overall reaction consuming O_2_(*aq*) can be thought of as proceeding in the following five steps: (1) Radicals K^•^ and Y^•^ are homogeneously produced from PA photolyzed in water. (2) The radicals, K^•^ and Y^•^, diffuse very rapidly to the water/air interface of bubbles. (3) The bimolecular reaction of dissolved O_2_ with adsorbed K^•^ and Y^•^ radicals generates their respective peroxyl radicals on the air bubble surface during the reactions:
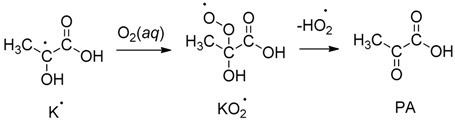
,(3)

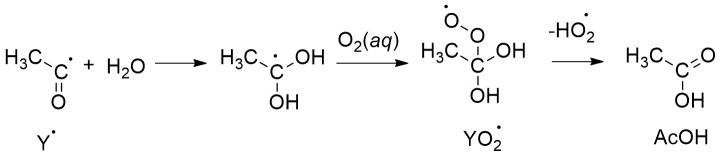
.(4)
The second order constants for the reactions of K^•^ and the hydrated Y^•^ radicals with O_2_(*aq*) are $k_{K^{•}+O_{2}}$ = 1.2 × 10^9^ M^−1^ s^−1^ and $k_{Y^{•}+O_{2}}$ = 7.7 × 10^8^ M^−1^ s^−1^, respectively [7,30], which are near the diffusion limit. Based on the mechanism previously published, the peroxyl radicals $\left({\mathrm{RO}}_{2}^{•}={\mathrm{KO}}_{2}^{•}+{\mathrm{YO}}_{2}^{•}\right)$ in the reactions above result in the regeneration of PA and the formation of acetic acid (AcOH), respectively [7]. (4) The photolysis products are desorbed from the surface of air bubbles. (5) The desorbed products quickly diffuse away from the surface of the air bubbles.

The reported reaction rates are useful to model the photolytic O_2_(*aq*) depletion that can occur in aqueous tropospheric aerosols. The zero-order half-life (*t*_t1/2_) of dissolved O_2_ in air saturated samples for the extremes of a dilute (10 mM) and a concentrated (200 mM) solution of PA reported in Figure 1 are $t_{1/2}^{10\;mM}$ = 22 and $t_{1/2}^{200\;mM}$ = 3 min, respectively. The range estimated arises from the different rates of radical photoproduction. For simplicity, the sum of the carbon centered radicals, K^•^ + Y^•^ = R^•^, can be considered to control the consumption of O_2_(*aq*) in a process that is faster than the mass transfer of oxygen to the solution (β) in Figure 1:

(5) $$\frac{-d[O_{2}\left(aq\right)]}{dt}=R_{-O_{2}\left(aq\right)}=\beta -k_{R^{∙}+O_{2}\left(aq\right)}\left[R^{∙}\right]\left[O_{2}\left(aq\right)\right]$$

For the experimental wavelength of relevance in these experiments, λ = 330 (± 25) nm, the carbon centered radicals are produced with photolysis rates of $J_{{\left[PA\right]}_{0}}{\left[PA\right]}_{0}=I_{0}\times \varepsilon_{\lambda }\times {\left[PA\right]}_{0}\times l\times \Phi_{-\mathrm{PA}}$ and mainly consumed in reactions with O_2_(*aq*):

(6) $$\frac{d\left[R^{∙}\right]}{dt}=J_{{\left[PA\right]}_{0}}{\left[PA\right]}_{0}-k_{R^{∙}+O_{2}\left(aq\right)}\left[R^{∙}\right]\left[O_{2}\left(aq\right)\right]$$

where the included molar absorption coefficient of PA, $\varepsilon_{\lambda }$, can be obtained from a solution of known concentration, *l* = 4.50 cm is the path length of irradiation for this reactor, the initial PA concentration is given by [PA]_0_, and the quantum yield of PA loss is *Φ*_–PA_ ≈ 2 [7]. The simplification introduced in Equation (6) considers that even for high [PA]_0_, reactions with O_2_(*aq*) can consume ≥ 90% of the carbon centered radicals, with the remaining ≤ 10% generating other products [7]. Future efforts for solving the complex system of the differential Equations (5) and (6) to provide numerical solutions should be possible by assuming the concentration of R^•^ remains the same during the reaction (in steady state [R^•^]*_ss_* = constant ⇒ *d*[R^•^]_ss_/*dt* = 0).

The following simple kinetic analysis assesses the importance of the reactions consuming O_2_(*aq*) to advance their understanding. From the extreme diluted to the most concentrated [PA]_0_ in Figure 2, the continuous photogeneration of K^•^ and Y^•^ is accelerated, enhancing the consumption of available O_2_(*aq*). Thus, under initial [O_2_(*aq*)] = 0.260 mM at 25 °C, the depth of penetration of O_2_ molecules into aqueous atmospheric particles can be estimated using the typical diffusion length equation,

(7) $$t\approx x^{2}/\left(2D_{O_{2}}\right)$$

and the estimated $t_{1/2}^{200\;mM}$ = 3 min and $t_{1/2}^{10\;mM}$ = 22 min of dissolved O_2_ during irradiation of 200 and 10 mM PA, respectively, under the conditions of Figure 1. The diffusion coefficient of O_2_ in water, $D_{O_{2}}=$ 2.20 × 10^−5^ cm^2^ s^−1^ [31], is needed to solve the distance travelled by molecules (*x*) from Equation (7). Thus, O_2_ molecules entering aqueous particles through the interface with air during photolysis of PA can easily travel as far as 1.6 to 4.3 µm during the time it takes for the photogenerated radicals to consume one half of the available dissolved O_2_. Consequently, only aqueous films thicker than 4 μm (or large coarse mode particles) that are also high in [PA] could be interpreted to undergo oxygen depletion under sunlight irradiation. Thus, only extreme conditions are capable of generating the fast reaction rates required to create O_2_(*aq*)-free environments until PA is exhausted. In other words, under relevant environmental conditions, the level of dissolved O_2_ in typical accumulation mode and even small coarse mode aqueous particles [32] should be easily replenished. However, at the microscopic level and based on the value of $k_{K^{•}+O_{2}}$ for a bimolecular process, a half of the O_2_ molecules dissolving into water and encountering K^•^ radicals should disappear within a few microseconds.

**Figure 3:**
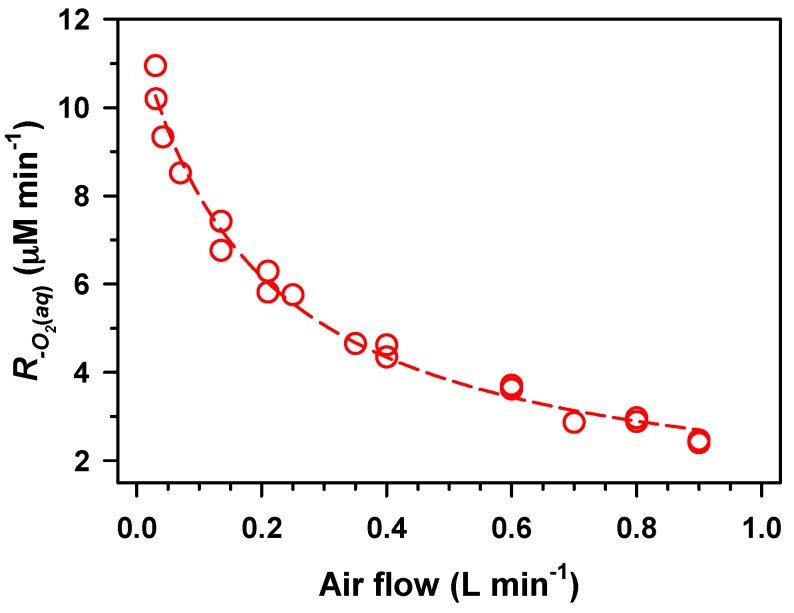
Rate of dissolved oxygen decay ($R_{-O_{2}\left(aq\right)}$) vs. variable air flows. Experimental conditions: [PA]_0_ = 20.0 mM at pH 1.0 and 25 °C irradiated at λ ≥ 305 nm (*I_a_* = 1.02 × 10^−5^ Einstein L^−1^ s^−1^). Represented reaction rates are multiplied by −1.

Figure 3 shows the dependence of $R_{-O_{2}\left(aq\right)}$ on the increasing flow rates of air through solutions of constant [PA] = 20.0 mM, for the same photon flux, pH, and temperature utilized in Figure 2. Thus, the variation of the reaction rates in Figure 3 is caused by the enhancement in the mass transfer of oxygen to the solution. As the flow rate of air increases in the experiments performed to collect the data for Figure 3, the [O_2_(*aq*)] levels off at a non-zero value much later than the times observed in Figure 1. It must be mentioned that all air flows in Figure 3 larger than 30 mL min^−1^ (the value used in Figure 1 and Figure 2) result in solutions that are not completely O_2_-depleted during the 1 h irradiation period. The use of zero-order kinetics ensures the data included in Figure 3 directly reflects how dissolved O_2_ is consumed. For higher continuous air sparging flow rates in Figure 3, the value of β is maximized. However, it must be noticed that the bubbling of the solution becomes too violent above 0.9 L min^−1^, which prevents any attempt to further increase the flow to make $R_{-O_{2}\left(aq\right)}\to$ 0. Specifically, the limit of $R_{-O_{2}\left(aq\right)}\to$ 0 would represent the point at which the mass transfer of O_2_(*g*) to the solution equals or exceeds the O_2_(*aq*) consumption by the carbon centered radicals generated by PA photolysis: β ≥ $R_{-O_{2}\left(aq\right)}$.

**Figure 4:**
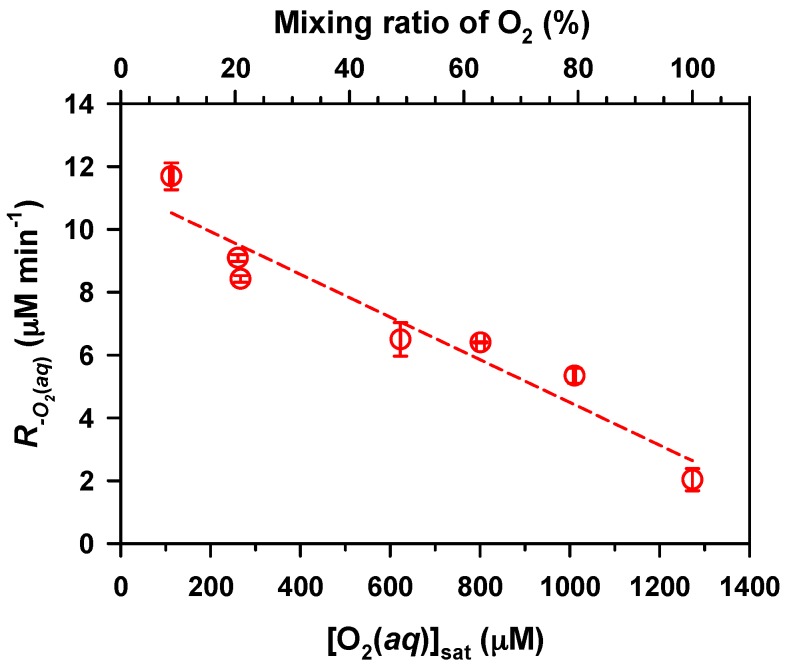
Rate of dissolved oxygen decay ($R_{{-O}_{2}\left(aq\right)}$) vs. dissolved oxygen concentration ([O_2_(*aq*)]) during irradiation (λ ≥ 305 nm, *I_a_* = 1.02 × 10^−5^ Einstein L^−1^ s^−1^) of 20.0 mM solutions of PA at pH 1.0 and 25 °C with continuous air sparging at 100.0 mL min^−1^ (incomplete oxygen depleted regime). For reference, the top horizontal axis represents the mixing ratio of O_2_(*g*) dissolved in N_2_(*g*). Represented reaction rates are multiplied by −1.

To further increase the mass transfer of oxygen to the solution and due to the clear limitations associated with simply increasing the flow rate of air, the next set of experiments uses a different gas source. Briefly, the sparging gas is prepared onsite by regulating the ratio of pure O_2_(*g*) and N_2_(*g*) in the mixing streams. In this way, the mixing ratio of O_2_(*g*) in the sparging gas could be controlled to reach the saturation levels expected in thermodynamic equilibrium. Figure 4 displays how $R_{-O_{2}\left(aq\right)}$ varies with the level of dissolved O_2_ under saturation conditions during the irradiation of [PA] = 20.0 mM utilizing a total gas flow of 100.0 mL min^−1^. For comparison, the actual mixing ratio of O_2_(*g*) employed is also indicated on the top axis of Figure 4. The experiments in Figure 4, varying the [O_2_(*aq*)] while keeping constant [PA]_0_ (and thus the production of R^•^), are helpful to determine the order of dissolved oxygen in the photoprocess. From the slope of the straight line fitting the data of Figure 4 (*m* = −6.87 × 10^−3^ min^−1^), it is calculated that for a 6-time drop in concentration (i.e., from 1200 µM to 200 µM O_2_(*aq*)), the corresponding decay rate of [O_2_(*aq*)] is 6-times slower. Therefore, the photoreaction of PA in water is first order in [O_2_(*aq*)]. Furthermore, these experiments achieve up to the theoretical saturation maximum for a 1 atm O_2_(*g*) in aqueous solutions of [O_2_(*aq*)] = 1240 μM. It must be noted that the experiments in Figure 4 using flows of 100 mL min^−1^ (> 30 mL min^−1^ discussed earlier when using air) are completed in a short enough time period that is impossible for the solutions to reach complete O_2_-depletion conditions during irradiation. A conclusion with atmospheric relevance is the impossibility of using 100% O_2_(*g*) to force the value of $R_{{-O}_{2}\left(aq\right)}$ to zero with the tools used. Figure 3 and Figure 4 demonstrate that neither increasing the gas flow rate nor the mixing ratio of O_2_(*g*) are effective ways to increase β enough to observe $R_{{-O}_{2}\left(aq\right)}$ = 0 in this setup.

**Figure 5:**
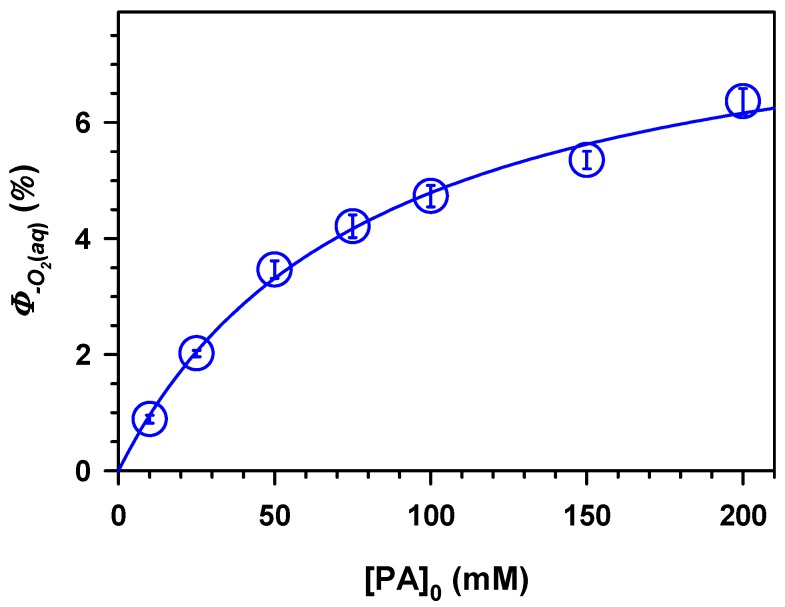
Quantum yield percentage of dissolved O_2_ loss ($\Phi_{{-O}_{2}\left(aq\right)}$) during the λ ≥ 305 nm irradiation of PA solutions at pH 1.0 and 25 °C with continuous air sparging at 30.0 mL min^−1^. The absorbed photon rates are provided in Figure 1.

Figure 5 displays the photochemical quantum yield of dissolved O_2_ loss ($\Phi_{-O_{2}\left(aq\right)}$) for the range of [PA]_0_ studied in the same experiments of Figure 2. The upper limit of $\Phi_{-O_{2}\left(aq\right)}$ = 8.64% is estimated from the extrapolation of the hyperbola to the limit case of [PA]_0_→ ∞, which is fitted with a coefficient of correlation of *r*^2^ = 0.993 to $\Phi_{-O_{2}\left(aq\right)}\left(\%\right)$ = 8.64 [PA]_0_/(80.3 + [PA]_0_). This hyperbolic behavior follows (as expected) the same kinetics described for the sum of the quantum yields of the photoproducts previously reported [7] and the Langmuir behavior of Figure 2. The larger likelihood for the addition of K^•^ to a molecule of PA for increasing [PA]_0_ than for the recombination of two K^•^ radicals forming 2,3-dimethyltartaric acid has been carefully described in the mechanism [7]. The hyperbolic behavior of Figure 5 can perfectly account for the faster growing branching ratio in the production of oxocarboxylic acids than 2,3-dimethyltartaric acid. Figure 5 and its matching hyperbolic behavior to the sum of the quantum yield of the products strongly supports the mechanism reported by Eugene and Guzman [7], which accounts for the effect of dissolved O_2_ to produce peroxyl radicals. The produced peroxyl radicals, however, cannot inhibit the production of higher molecular weight dimers and trimers of PA.

## 3. Materials and Methods

### 3.1. Preparation of Experiments

**Figure 6:**
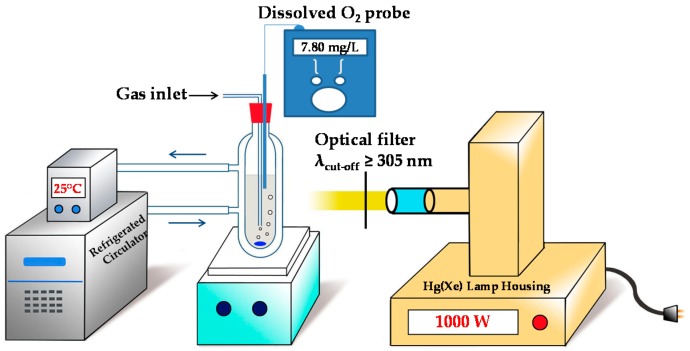
Diagram of the photochemical setup.

Each reaction was performed in duplicate. Fresh solutions of PA (Sigma-Aldrich, Milwaukee, WI, USA, 98.5%), freshly distilled under vacuum in ultrapure water (18.2 MΩ cm^−1^, Elga Purelab Flex, Veolia, Paris, France) were prepared immediately before each photolysis. The choice of working concentrations was described in detail previously; briefly, it was estimated that acidic, urban aerosols may contain 5–200 mM PA [7,23,24]. The pH of solutions was adjusted by adding hydrochloric acid (EMD, Gibbstown, NJ, USA, 37.7%) or sodium hydroxide (Amresco, Solon, OH, USA, 99.0%) and measured using a calibrated pH electrode (Orion, Thermo Scientific, Beverly, MA, USA). A 200 mL aliquot of the pH adjusted solution was transferred to a homemade quartz photochemical reactor (University of Kentucky Glass Shop, Lexington, KY, USA) with a capacity of 220 mL. This photochemical reactor was equipped with a jacket for temperature control as depicted in Figure 6 and described previously [33,34]. The solution in the photoreactor was sparged in the dark with air, O_2_(*g*), N_2_(*g*), or a custom O_2_(*g*)/N_2_(*g*) mix through a Teflon tube with an internal diameter of 1.6 mm starting 30 min prior to photolysis. The corresponding calculated diameter of the detached bubbles was *ca.* 4.1 mm [35]. As the sparging of the desired gas continued during the course of the experiments, the headspace of the photoreactor was filled with the selected gas during the complete course of the reaction. For experiments with variable O_2_(*g*) mixing ratios, streams of pure N_2_(*g*) and O_2_(*g*) were mixed as needed with a gas proportioner (Cole-Parmer, Vernon Hills, IL, USA) utilizing a total flow of 100 mL min^−1^. Monitoring of [O_2_(*aq*)] during the reactions was provided by a polarographic oxygen probe (Thermo Orion, Beverly, MA, USA, 081010MD) calibrated at maximum saturation in an air-calibration sleeve and at 0% saturation in a saturated sodium sulfite/CoCl_2_ solution.

### 3.2. Photochemical Conditions

The solutions were irradiated with a 1 kW Xe-Hg lamp filtered through (1) water and (2) a cutoff filter (Newport, Irvine, CA, USA) at λ ≥ 305 nm (Figure 6) [7,8,9,26,33,34,36,37]. The effective incident photon flux of the lamp, *I*_0_, reported was obtained after correcting the actinometric measurement by comparing the convoluted spectra of both phenylglyoxylic acid and PA with the lamp. The reported actinometer quantum yield of CO_2_ production was $\Phi_{{\mathrm{CO}}_{2}}$ = 0.39 at pH 3.0 and 25 °C [38,39]. The concerted photodecarboxylation of 5.0 mM phenylglyoxylic acid by the reaction:

PhC(O)CO_2_H + *hν* → PhC(O)C(O)H + CO_2_(*g*) (8)

was monitored by FTIR spectroscopy (iZ10 FTIR module connected to Thermo Scientific Nicolet iN10 infrared microscope) to register the rate of CO_2_(*g*) evolution. The use of 5.0 mM phenylglyoxylic acid (Sigma, St. Louis, MO, USA, ≥98.0%) as an actinometer was convenient due to its similar absorption to a 100.0 mM PA in the near UV spectrum. There was no traceable difference in the tails of both spectra from 355 to 420 nm. Furthermore, the correction applied using the convoluted spectra with the lamp ensures the quantum yields reported in these experiments are valid in the narrow band of λ = 330 (± 25) nm. The experimentally determined effective photon flux, *I*_0_ = 1.13 (0.03) × 10^−5^ Einstein L^−1^ s^−1^, was used to calculate the quantum yield of dissolved O_2_ loss, $\Phi_{-O_{2}\left(aq\right)}$, by dividing the corresponding reaction rate in the absorbed photon flux, *I_a_*, which is obtained from:

(9) $$I_{a}=I_{0}\left(1-e^{-2.303\;\varepsilon \;l\;{\left[\mathrm{PA}\right]}_{0}}\right),$$

where *ε* = 11.3 M^−1^ cm^−1^ is the molar absorption coefficient of PA at λ_max_ = 321 nm, *l* = 4.50 cm is the path length of irradiation for this reactor; and the initial PA concentration is given by [PA]_0_.

Equation (9) stipulates that light absorption and therefore the photolysis rate of this reaction varies linearly [7] with [PA]_0_ only below ~1 mM for *l* = 4.5 cm (originally explained by Guzman et al. for *l* = 1 cm to be [PA]_0_ < 4 mM) [23]. The previous important concept [7,23] must be taken into account in any relevant photochemistry study of PA. However, for 1 < [PA]_0_ < 300 mM, the photolysis rate must decrease as the rate of photon absorption drops. Therefore, PA loss approaches a maximum for the case of a high enough solution absorbance, or in other words, when *I*_0_ = *I_a_*. From Equation (9), the absorbed photon rates are *I_a,10 mM_* = 7.80 × 10^−6^ Einstein L^−1^ s^−1^ for [PA]_0_ = 10 mM; *I_a,25 mM_* = 1.07 × 10^−5^ Einstein L^−1^ s^−1^ for [PA]_0_ = 25 mM; and *I_a,50-200 mM_* = 1.13 × 10^−5^ Einstein L^−1^ s^−1^ for 50 ≤ [PA]_0_ ≤ 200 mM. During irradiation, the solutions were maintained at 25 °C and magnetically stirred while being continuously sparged with the desired gas.

## 4. Conclusions

The results presented are of general atmospheric chemistry interest due to the relevance that radical reactions play in aerosol droplets. The contribution of aqueous phase photochemistry to the generation of secondary organic aerosol has been recently recognized as important and different from the gas phase processing of volatile organic compounds [40,41]. Particularly, the complex photoproducts of low volatility from the aqueous phase photochemistry of PA can remain in the particle phase, potentially affecting aerosol properties such as their absorption, scattering, morphology, and hygroscopicity, which themselves alter the atmospheric residence time of particles [42]. This laboratory study has shown the importance of properly controlling the mass transfer of O_2_(*g*) into water, which plays a major role in the processing of soluble organic matter by creating peroxyl radicals [7,23,28]. The rate-determining step of the entire process studied here is controlled by an interfacial reaction, occurring on an interface that becomes saturated with the reactant following a Langmuir isotherm. The study of the photophysics of the elementary steps of the reaction by time resolved measurements would be beneficial to advance this work further.

The work has provided a detailed understanding of the rate of consumption of dissolved O_2_ during the photochemistry of PA under variable flow rates of air, reactant concentrations, and initial saturated [O_2_(*aq*)] for equilibria. These observations are atmospherically relevant for any study that would like to consider (and reconsider) the radicals from PA photochemistry to initiate the photooxidative processing of biogenic and anthropogenic emissions [32]. For example, the degradation of isoprene derived [MVK] = 20 M was triggered by irradiated [PA] = 100 mM and analyzed after 50 min to characterize the oligomers formed [28]. However, the experimental analysis at 50 min clearly reflects the presence of large oligomers as heavy as 1200 Da, which could only be produced as a consequence of working under depleted O_2_(*aq*) conditions (reported as [O_2_(*aq*)] = 12 µM) [28], which are not atmospherically relevant. The lack of dissolved O_2_ in the aqueous solutions greatly affects the rate and extent of oligomerization. Thus, it is recommended that the conditions for such relevant experiments are optimized by monitoring the [O_2_(*aq*)] while varying, i.e., the flow rate, reactant concentrations, mixing ratios, etc., as illustrated above.

Overall, the work contrasts oxic and anoxic regimes during the photochemistry of aqueous PA, and the results serve as a reference point to characterize systems operating under limited conditions for oxygen mass transfer to the solution. Future studies are needed to reassess the effect of the photogenerated ketyl and acetyl radicals in the processing of isoprene derived water-soluble compounds under relevant oxic conditions existing in the atmosphere.
